# Nasojejunal tube, jejunostomy tube, and fine needle catheter jejunostomy effects after esophagectomy

**DOI:** 10.3389/fsurg.2026.1777414

**Published:** 2026-05-20

**Authors:** Wu Wang, Tianbao Yang, Jinbiao Xie, Shijie Huang, Boyang Chen

**Affiliations:** Department of Cardiothoracic Surgery, The Affiliated Hospital of Putian University, Putian, China

**Keywords:** esophagectomy, feeding method, fine needle catheter jejunostomy, jejunostomy tube placement, nasojejunal tube placement

## Abstract

**Introduction:**

To analyze and compare the effect of nasojejunal tube placement (NTP), jejunostomy tube placement (JTP), and fine needle catheter jejunostomy (FNCJ) after esophagectomy.

**Methods:**

Herein, 159 patients with esophageal carcinoma who underwent esophagectomy were retrospectively analyzed and divided into the following groups: the NTP group (*n* = 68), JTP group (*n* = 51), and FNCJ group (*n* = 40). The operation time, complication rate, average time to start a liquid diet, and average length of hospital stay were compared among the three groups.

**Results:**

The complication rate was significantly higher in the NTP group than in the two other groups (*P* < 0.05), and it did not statistically significantly differ between the JTP and FNCJ groups (*P* > 0.05). The tube placement time and average length of hospital stay were also significantly longer in the NTP group than in the two other groups (*P* < 0.05). The NTP group was associated with significantly longer tube placement time, average length of hospital stay, and average time to start a liquid diet (*P* < 0.05). The operation time was significantly longer in the JTP group than in the FNCJ group (*P* < 0.05). The average time to start a liquid diet and length of hospital stay were slightly longer in the JTP group than in the FNCJ group (*P* > 0.05).

**Conclusions:**

NTP showed distinct advantages and disadvantages compared with JTP and FNCJ. When selecting the feeding method, comprehensive consideration should be given to the patient's specific condition.

## Graphical Abstract

**Figure:**
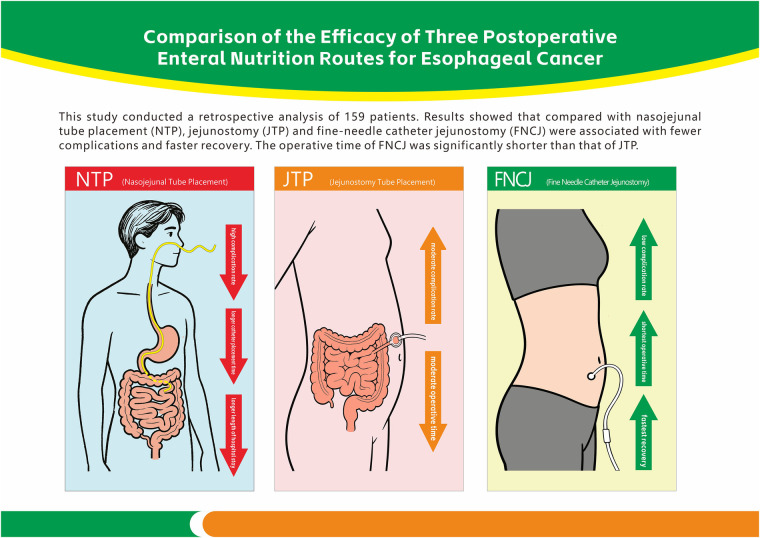

## Introduction

Esophageal cancer (EC) is among the most common malignant tumors in the world, particularly in north-central China, and it typically has high morbidity and poor outcomes (1, 2). Surgery, a radical tumor treatment, is among the most effective treatments for EC (3). With continuous advancements in surgical techniques, various methods of EC treatment have emerged, such as esophagogastrostomy and esophageal reconstruction (4). Although these methods have high success rates, there are some issues that cannot be ignored, such as large trauma and multiple postoperative complications like anemia and hypoproteinemia (5, 6). Moreover, patients cannot eat orally for 7–9 days postoperatively, and even after that, they face difficulty eating for a long time, which may lead to poor nutritional status and even cause an anastomotic leak (7). Therefore, the physical condition and nutritional support of postoperative patients are essential for successful surgery and rapid recovery of the patient (8).

Early enteral nutrition can improve the nutritional status and provide a better prognosis than parenteral nutrition (9). In recent years, nasojejunal tube placement (NTP), jejunostomy tube placement (JTP), and fine needle catheter jejunostomy (FNCJ) have been successfully applied in esophagectomy (10). Nevertheless, these methods have distinct characteristics and risks (10–12). NTP is a common method used by most medical centers; however, it shows poor comfort and tolerance in many cases. Jejunostomy can avoid the above shortcomings, but it is a more invasive procedure and can induce tube-related complications like intestinal obstruction. Therefore, we selected EC patients who underwent esophagectomy and investigated the application effects of the three routes of enteral nutrition to provide a reference for clinicians to select the appropriate mode of nutritional supplementation.

## Materials and methods

### General information

Herein, we retrospectively analyzed a total of 159 EC patients having undergone esophagectomy in the Affiliated Hospital of Putian University from January 2017 to November 2022. We included patients (1) with preoperative stages 0–III, (2) with availability of complete clinical data, and (3) having undergone no preoperative radiotherapy, chemotherapy, or other treatments. We excluded patients with (1) poor pulmonary function and cardiac dysfunction, (2) contraindications to NTP, JTP, or FNCJ, and (3) distant metastasis. The present study was approved by the Ethics Committee of our hospital and complied with the Declaration of Helsinki. The informed consent was obtained from all participants. In this study, time to start oral liquid diet was defined as the duration from the day of surgery to the first day of oral intake after confirmation of anastomotic healing by esophagography (routine day 7). Oral intake was not initiated until anastomotic healing was confirmed. For patients without confirmed healing (e.g., anastomotic leakage), oral intake was delayed until repeat esophagography confirmed adequate healing. Length of hospital stay was defined as the duration from the day of surgery to the day of discharge.

### Method

The thoracic stage was completed via right thoracoscopy. All patients underwent a three-incision McKeown esophagectomy with cervical anastomosis. The gastric conduit was created laparoscopically and pulled up to the neck through the posterior mediastinal route. Before surgery, patients were given single-lumen endotracheal intubation with intravenous anesthesia and placed in the supine position, and their head and left abdomen were raised by 15–30 degrees. The laparoscopic surgery was performed using the 5-port method. First, the skin was transversely incised 1 cm below the umbilicus, and an artificial pneumoperitoneum was created using the Veress needle, with an intra-abdominal pressure of 12 mmHg to establish a 10-mm observation hole. A surgical incision was created 2 cm above the umbilicus on both sides of the body, lateral to the respective midclavicular line. At the junction of the right axillary line and the right costal margin of the patient, the main operating hole was established on the right hand of the operator; a 10-mm operating hole was built on the left hand of the operator, and a 5-mm operating hole was established under the xiphoid process. Next, laparoscopic mobilization of the greater and lesser curvature of the stomach, lymph node dissection, and abdominal esophageal mobilization were performed. A tubular stomach was created laparoscopically. The diaphragmatic hiatus was then incised to allow adequate mobilization of the gastric conduit, followed by jejunostomy tube placement.

For JTP, the transverse colon 20 cm from the ligament of Trendelenburg was marked and lifted to the tip, and a hole in the intestinal wall was opened at the contralateral edge of the mesentery below it as the puncture stoma point. Around the puncture point, an absorbable suture was used to preset the “half purse-string” for three consecutive stitches; the absorbable suture was extracted from the left surgical hole, and the “half purse-string” on the other side was preset. Then, the Seldinger method was used to insert the trocar from the auxiliary surgical hole on the left side, penetrate the intestinal wall, and establish the fistula in the distal jejunum. Next, the feeding tube was passed through the trocar and extended into the distal jejunal lumen. Outside the abdominal cavity, we gently tightened the “semi purse-string” line, slowly injected saline, and inserted and advanced the feeding tube ∼40 cm while ensuring that it was not twisted or tangled. Subsequently, physiological saline was injected to check the patency of the catheter and confirm the position of the end of the fistula tube. A “semi purse-string” line was used to stabilize the fistula. Finally, the feeding tube was secured to the abdominal wall from outside the body, ensuring that the jejunum around the fistula did not form a sharp angle to proceed with intra-abdominal manipulation.

#### NTP

The feeding tube was slowly introduced in and advanced through the nasal cavity, passed through the esophagus and advanced into the stomach until the Treiz ligament was reached; once the length of the feeding tube reached 80 cm, the tube was not advanced any further. The feeding tube was fixed and held in place outside the nostrils with a tape.

FNCJ was performed using a fine needle catheter jejunostomy device and jejunostomy tube. While the patient lay supine on the bed, the operator made a 1-cm incision 5 cm above the left mid-clavicular line at the navel. At the same time, small 0.5-cm incisions below the xiphoid process and bilateral rib margins were made to assist with the operation. Mobilization and tubular creation of the stomach were laparoscopically performed, and an incision of ∼0.5 cm was made near the predetermined jejunostomy site, at 30 cm from the ligament of Treitz, reaching the deep fascia layer, indicating the location of the stoma. Then, using a 3-0 suture, purse-string sutures were performed on the semicircular layer of the proximal jejunal wall, and both ends of the suture were extracted from the body through a skin incision using a puncture hook. Under the tension applied by the assistant, the jejunum was lifted and obliquely pierced into the center of the semi-purse string with a detachable trocar. Then, the sutures were tightened, and the distal bowel was oriented, allowing the puncture needle to penetrate 1.5–2 cm beneath the muscular layer before entering the intestinal lumen.

### Indicators

The tube placement time, complication rate, time to start a liquid diet, and average length of hospital stay were compared among the three groups.

### Statistical methods

All data were analyzed using SPSS24.0 software. One-way ANOVA and t-test were used for inter-group comparisons, with homogeneity of variance test (Leven test) and normality test (*F*-test) performed for the analysis of each group of data. Normally distributed measurement data were expressed as the mean ± standard deviation, and non-normally distributed measurement data were expressed as the median (interquartile range). Enumeration data were statistically analyzed by the chi-square test, with *P* < 0.05 considered statistically significant.

## Results

### Baseline data

According to different uses of jejunostomy tubes, patients were divided into the NTP group (*n* = 68), JTP group (*n* = 51), and FNCJ group (*n* = 40). As shown in Table 1, their general information was similar and comparable (*P* > 0.05). In the NTP group, there were 37 men and 31 women (average age, 58.79 ± 5.75 years). All patients were preoperatively diagnosed with EC, including 66 cases of squamous cell carcinoma, 1 case of sarcomatoid carcinoma, and 1 case of adenocarcinoma. Pathological stage distribution was as follows: stage 0, 1 case; stage I, 16 cases; stage IIA, 30 cases; stage IIB, 6 cases; and stage IIIA, 15 cases.

**Table 1:** Baseline data of the three groups of patients.

Group	N	Male (n)	Female (n)	Average age (years)	Stage 0	Stage I	Stage IIA	Stage IIB	Stage IIIA
NTP group	68	37	31	58.79 ± 5.75	1	16	30	6	15
JTP group	51	28	23	59.13 ± 5.64	1	13	23	5	9
FNCJ group	40	22	18	58.14 ± 5.68	1	10	18	4	7

NTP, nasojejunal tube placement; JTP, jejunostomy tube placement; FNCJ, fine needle catheter jejunostomy.

The JTP group comprised 28 men and 23 women (average age, 59.13 ± 5.64 years), including 49 cases of squamous cell carcinoma, 1 case of sarcomatoid carcinoma, and 1 case of adenocarcinoma. Pathological stage distribution was as follows: stage 0, 1 case; stage I, 13 cases; stage IIA, 23 cases; stage IIB, 5 cases; and stage IIIA, 9 cases.

The FNCJ group comprised 22 men and 18 women (average age, 58.14 ± 5.68 years), including 38 cases of squamous cell carcinomas, 1 case of sarcomatoid carcinoma, and 1 case of adenocarcinoma. Pathological stage distribution was as follows: stage 0, 1 case; stage I, 10 cases; stage IIA, 18 cases; stage IIB, 4 cases; and stage IIIA, 7 cases.

### Comparison of the complication rate among three groups after esophagectomy

As shown in Table 2, the complication rate was significantly higher in the NTP group than in the other two groups (*P* < 0.05). However, there was no statistically significant difference in the complication rates between the JTP and FNCJ groups (*P* > 0.05).

**Table 2:** Comparison of the complication rate.

Group	N	Anastomotic leak	Anastomotic stricture	Gastrointestinal reflux	Pulmonary infection	Complication rate (%)
NTP group	68	2	5	5	4	16 (23.5)
JTP group	51	1	2	3	2	8 (15.7)
FNCJ group	40	1	1	2	1	5 (12.5)
X^2^						5.027
*P*						0.027

NTP, nasojejunal tube placement; JTP, jejunostomy tube placement; FNCJ, fine needle catheter jejunostomy.

### Comparison of tube placement time, time to start a liquid diet, and average length of hospital stay among three groups

As shown in Table 3, the tube placement time, the average length of hospital stay, and the average time to start a liquid diet were all significantly longer in the NTP group than in the two other groups (*P* < 0.05). The operation time was significantly longer in the JTP group than in the FNCJ group (*P* < 0.05). The average time to start a liquid diet was slightly longer in the JTP group than in the FNCJ group (*P* > 0.05).

**Table 3:** Comparison of tube placement time, time to start a liquid diet, and average length of hospital stay.

Group	n	Tube placement time (min)	Average time to start a liquid diet (days)	Average length of hospital stay (days)
NTP group	68	20.5 ± 3.7	14.6 ± 2.3	16.3 ± 5.2
JTP group	51	17.3 ± 2.4	11.9 ± 1.5	13.8 ± 4.7
FNCJ group	40	14.8 ± 1.6	11.4 ± 1.3	12.4 ± 4.1
*F*		51.36	49.39	8.64
*P*		0.001	0.001	0.001

NTP, nasojejunal tube placement; JTP, jejunostomy tube placement; FNCJ, fine needle catheter jejunostomy. Time to start a liquid diet was defined as the duration from the day of surgery to the first day of oral intake after confirmation of anastomotic healing by esophagography (routine day 7). Oral intake was not initiated until anastomotic healing was confirmed. For patients without confirmed healing (e.g., anastomotic leakage), oral intake was delayed until repeat esophagography confirmed adequate healing. Length of hospital stay was defined as the duration from the day of surgery to the day of discharge.

## Discussion

This study showed that the complication rate was higher in the NTP group than in the other two groups. However, it did not statistically significantly differ between the two other groups. Notably, nasojejunal tube implantation is a relatively simple method, which involves inserting a slender tube through the nasal cavity into the esophagus and advancing it until its end reaches the jejunum. Nevertheless, the study showed NTP had no advantages on shortening the tube placement time, the average length of hospital stay, and the average time to start a liquid diet. The nasojejunal tube is commonly used in the early postoperative period for short-term nutritional support (13, 14). However, its insertion has some drawbacks. As the catheter is inserted via the nasal cavity and esophagus, patients may feel uncomfortable and have a foreign body sensation, leading to swallowing difficulties and sore throat (12). In addition, it may cause increased irritation and mucous secretion in the nasopharynx, which is not conducive to coughing. The tube is also prone to accidental removal or detachment by patients, which is not conducive to long-term nutritional support. Finally, long-term use of nasojejunal tubes can lead to trauma and infection of the nasal cavity and esophagus (15). Regarding anastomotic stricture, although our study only reports an association between NTP and a higher incidence of stricture, a possible mechanistic explanation is that passing the NTP tube through the entire length of the gastric conduit and the cervical anastomosis may cause local irritation or micro-trauma at the anastomotic site, potentially promoting scar formation and subsequent stricture. In contrast, JTP and FNCJ deliver nutrients distal to the anastomosis, avoiding any mechanical interaction with the anastomotic site. Further studies are needed to confirm this hypothesis.

Jejunostomy involves inserting a catheter connected to one end of the jejunum through abdominal surgery, with the other end exposed to the abdominal wall for the delivery of nutrients. This method can provide long-term nutritional support, and patients can avoid discomfort caused by nasojejunal tubes (16). However, it is relatively complex in terms of surgery and postoperative care and requires patients to maintain good pipeline cleanliness during recovery.

FNCJ is a relatively new method that involves inserting a fine needle catheter into the jejunum under endoscopic guidance for surgical intervention in the abdomen; the other end of the catheter is exposed to the abdominal wall for nutrient delivery. Unlike traditional jejunostomy, this method involves puncturing the abdominal and jejunal walls, resulting in less trauma and reduced risk of intestinal fluid leakage (17). Therefore, fine needle catheter jejunostomy has a relatively lower incidence of complications as it greatly avoids most complications that typically occur in most abdominal surgeries, such as traumatic infection and postoperative pain. However, it requires endoscopic guidance and small incisions in the abdominal area, which may lead to some postoperative complications. Therefore, NTP is a simpler method for nutritional support after EC resection; however, the incidence of complications is high, particularly with its long-term use, which can easily lead to trauma and infection of the nasal cavity and esophagus (10). Conversely, although JTP provides long-term nutritional support, the surgery and postoperative care are complex and require patients to maintain good pipeline cleanliness to prevent complications. Finally, FNCJ is a relatively new method, which has less trauma compared with traditional jejunostomy and does not require major abdominal surgery, thus reducing the risk of complications (17).

The process of inserting a nasojejunal tube is relatively simple; however, owing to the complex position of the tube inserted through the nasal cavity into the esophagus, the insertion process can take a long time to complete (18). For patients with confirmed anastomotic leakage, oral feeding was delayed until repeat esophagography confirmed adequate healing. Furthermore, compared with the other two approaches, the time to start a liquid diet after NTP is relatively late, and the patients have to wait longer to receive nutritional support during the postoperative recovery process. Moreover, owing to the high incidence of complications in patients undergoing NTP, the recovery process after surgery requires a longer time, resulting in longer length of hospital stay. Conversely, JTP requires abdominal surgery to insert the tube into the jejunum, which is relatively complex and requires a longer tube placement time. Furthermore, owing to the complexity of the surgery, patients need more time to recover, resulting in delay in starting a liquid diet. In terms of average hospitalization time, similar to NTP, the complexity of jejunostomy leads to longer hospital observation and care. Compared with jejunostomy, FNCJ is a relatively new method, which does not require additional laparotomy, and thus, the catheterization time is relatively short. Owing to the shorter catheterization time and time to start a liquid diet, patients can receive sufficient nutritional support earlier after surgery. Compared with the other two methods, FNCJ causes less surgical trauma and shows fast recovery, resulting in a shorter average length of hospital stay. Therefore, the FNCJ performed well in terms of catheterization time, average time to start a liquid diet, and length of hospital stay because it did not require open surgery and showed shorter catheterization time. With this approach, patients can receive fluid and nutritional support earlier, thus accelerating recovery and minimizing the average length of hospital stay (19). Therefore, both JTP and NTP have similar performance, with both requiring longer tube placement time, longer time to start a liquid diet, and relatively longer average hospitalization time than FNCJ. Notably, the specific esophagectomy technique—including the site of anastomosis (cervical *vs*. intrathoracic), the surgical approach (minimally invasive *vs*. open), and the extent of gastric conduit reconstruction—may affect tube placement difficulty and postoperative tube retention time. For example, cervical anastomosis may increase the risk of nasojejunal tube displacement, whereas a minimally invasive approach may facilitate more precise FNCJ placement and shorten the procedure time. Furthermore, different surgical techniques have varying effects on gastric emptying; patients with delayed gastric emptying may require prolonged enteral nutrition regardless of the feeding tube type. The present study did not stratify patients by surgical technique, which could be a potential confounding factor. We acknowledge that the hospital stay in our study (NTP: 16.3 ± 5.2 days; JTP: 13.8 ± 4.7 days; FNCJ: 12.4 ± 4.1 days) is longer than that reported in Western ERAS centers (7-9 days), which is a limitation. This may be attributed to the absence of routine ERAS protocols, a higher complication rate (particularly in the NTP group), and the routine use of esophagography on postoperative day 7 in our study before initiating oral intake. In addition, differences in healthcare systems between China and Western countries may also contribute to this discrepancy. In China, patients often prefer to remain in the hospital until complete symptomatic recovery and confirmation of adequate oral intake without complications, whereas in many Western ERAS centers, discharge criteria emphasize functional recovery (e.g., adequate oral intake, pain control with oral medication, and mobilization) rather than complete resolution of all symptoms. Furthermore, routine esophagography on postoperative day 7, which is standard at our institution, may prolong hospitalization compared to Western centers that omit routine imaging or perform it earlier.

## Conclusion

In summary, NTP, JTP, and FNCJ showed distinct advantages and disadvantages in patients having undergone esophagectomy. When selecting the surgical procedure, comprehensive consideration should be given to the patient's specific condition and nutritional status.

## Data Availability

The raw data supporting the conclusions of this article will be made available by the authors, without undue reservation.
